# Efficacy of music therapy in patients with mild cognitive impairment. Systematic review

**DOI:** 10.1192/j.eurpsy.2025.1750

**Published:** 2025-08-26

**Authors:** I. Rodríguez Blanco, L. M. D.-P. Barros, P. S. Aranda, C. G. Cerdán, M. L. Argudo, R. K. G. Bolaños, C. M. Cossio, R. M. B. Rey

**Affiliations:** 1 University Clinical Hospital of Salamanca, Psychiatry, Salamanca, Spain

## Abstract

**Introduction:**

The concept “mild cognitive impairment” (MCI) means a decline in executive functions (such as memory, attention, language or thought) that does not correspond to what is expected for a person’s age group. It is estimated that this diagnosis may affect a fifth of the population over 65 years and 50-80% of them will develop dementia. This pathology is related to a loss of autonomy and an increase in dependence. In addition, there are therapeutic limitations, so it is a flagrant health and social problem. In this context of difficulties, various non-pharmacological therapies are emerging with the aim of improving various aspects of this disease, among which we can found music therapy (MT).

**Objectives:**

The aim of this study is to review the most recent findings of the scientific community regarding the validity of MT as an intervention in patients with MCI. Specifically, its efficacy on cognition and its power to stop the progression of dementia are evaluated, as well as its effects on other areas of the patient.

**Methods:**

A systematic review was carried out in the “WOS-Web of Science”, “Scopus” and “Psycoinfo” databases following PRISMA guidelines. The keywords were “MT” and “MCI”. We included clinical trials, systematic reviews and meta-analyses in english or spanish whose study population had MCI, excluding those published before 2017.

**Results:**

15 studies were selected, all with high quality evidence designs measured by Scotish Intercollegiate Guidelines Network scale. Among all the types of MT evaluated, various studies agree that Active MT (which includes activities that involve active participation of the patient such as singing or dancing) stands out as one of the best options, showing post-intervention improvements in MMEE scores and, secondarily, in emotional well-being (specially depression and anxiety). Instrumental practice has an important protective effect on cognitive function. On the other hand, MT with movement, in addition to being effective on cognition (it increases activity in prefrontal cortex), also causes an improvement in physical conditions. However, musical reminiscence (which consists of listening to music with an emotional component for patients with simultaneus display of images to favor memories), although it shows postive effects in several articles, these are not statistically significant. Finally, multimodal therapy (which is a combination of training and cognitive stimulations based on reminiscence and MT) did not show statistically significant changes in either mood or executive functions.

**Conclusions:**

MT is a valid intervention to improve cognitive function, some neuropsychiatric symptoms and the quality of life of patients with MCI. If we also take into account its economic accessibility, the organizational simplicity and null adverse effects, it is easily concluded to be one of the most attractive therapeutic options for treating MCI today.

**Disclosure of Interest:**

None Declared

